# Acupoint catgut embedding for obesity: A protocol of systematic review

**DOI:** 10.1097/MD.0000000000023728

**Published:** 2020-12-18

**Authors:** Wei Huang, Xia Chen, Yanji Zhang, Lihua Wang, Jiajie Wang, Yingrong Zhang, Dan Wei, Zhongyu Zhou

**Affiliations:** a Hubei University of Chinese Medicine/The Co-innovation Center for Preventive Treatment of Disease of Acupuncture-moxibustion in Hubei Province; b Department of Acupuncture, Hubei Provincial Hospital of Traditional Chinese Medicine, Wuhan, China.

**Keywords:** acupoint catgut embedding, acupuncture, obesity, study protocol, systematic review

## Abstract

**Background:**

Obesity is a chronic metabolic disease in which patients are overweight due to the excessive accumulation of fat in the body. As a subtype of acupuncture, catgut embedding at acupoints has increased in clinical application for obesity. The aim of this study is to evaluate the effectiveness and safety of acupoint catgut embedding therapy for simple obesity.

**Methods and analysis:**

Electronic searches of the Cochrane Library, PubMed, Springer Medline, EMBASE, Web of Science, China National Knowledge Infrastructure (CNKI), Wan-Fang Data (WANFANG), Chinese Biomedical Literature Database (CBM), and Chinese Scientific Journal Database (VIP databases) will be performed. The Chinese Clinical Trial Registry Center and the ClinicalTrials.gov registry will also be searched for ongoing trials. Databases will be searched from inception to August 2020.Randomized controlled clinical trials (RCTs) will be included if acupoint catgut embedding was evaluated as the sole treatment (diet or exercise therapy as the control group will be allowed) for simple obesity. The primary outcomes will consist of the improvement rate and reduction in body weight (BW). The secondary outcomes will include body mass index (BMI), waist circumference (WC), fat percentage (F %) and adverse effects. Two reviewers will undertake the study selection, data extraction and assessments of study quality. After screening the studies, the quality of the included studies will be assessed according to the quality criteria specified by the Cochrane Handbook for Systematic Reviews of Interventions (version 5.1.0). Meta-analysis will be performed by RevMan 5.3 software.

**Results:**

According to the data of improvement rate and reduction in BW, BMI, WC, and F %, this study will provide an evidence-based review of acupoint catgut embedding therapy for simply.

**Conclusions:**

This systematic review will present the current evidence for acupoint catgut embedding therapy for obesity.

**Ethics and dissemination:**

Ethical approval is not necessary since this protocol is only for systematic review and does not involve privacy data. The findings of this study will be disseminated electronically through a peer-review publication or presented at a relevant conference.

**Trial registration number:**

INPLASY2020110045.

## 1 Introduction

Obesity is a chronic metabolic disease in which patients are overweight due to the excessive accumulation or abnormal distribution of fat in the body.^[1]^ Simple obesity is defined as over-nutrition without an obvious cause wherein the weight of the patient exceeds the standard weight due to overabundant body fat when the accumulation of body fat exceeds the consumption level of the body.^[2]^ Along with the development of the modern social economy and changes in lifestyle, the prevalence of obesity is increasing in both developed and developing countries.^[3–5]^

According to epidemiology data, from 1980 to 2008, the average body mass index (BMI) increased by 0.4 kg/m^2^ every 10 years for both men and women worldwide.^[6]^ Over those 28 years, the global population of obese individuals increased from 6.4% to 12.0%, and the overweight population increased from 24.6% to 34.4%.^[7]^ If these trends continue, 60% of the global population, i.e., 3.3 billion people, will be overweight or obese by 2030.^[8]^ Reports have indicated that obesity can cause hypertension, diabetes, cardiovascular diseases, metabolic diseases, malignant tumors, and other conditions that increase cardiovascular and metabolic risks and contribute to premature death.^[9,10]^

Acupuncture, which originated in China and is based on the framework of traditional Chinese medicine, is an important alternative and complementary therapy for treating numerous diseases such as obesity. As an available and affordable treatment choice, acupuncture is popular with many patients and has been extensively used worldwide.^[11–13]^ When performed by qualified acupuncturists, it often has an excellent safety profile, and serious adverse events are rare.^[14]^

As a subtype of acupuncture, catgut embedding at acupoints has increased in clinical application in China during recent years. Continuous development of this treatment has occurred over the last 60 years, and it has been shown to have important effects on chronic diseases in many clinical studies.^[15,16]^ Currently, catgut embedding at acupoints is a popular intervention used to treat patients with simple obesity.^[17,18]^ In the treatment, bioprotein catgut is embedded into acupoints and stimulates them over a long-time period, exerting continuous effects. Compared with standard acupuncture, the advantages of catgut embedding at acupoints arise from its longer effects, higher patient compliance, and stronger long-term effects.^[19]^ Moreover, when treating simple obesity, catgut embedding has beneficial effects on dysmenorrhea, hyperglycemia, hyperlipidemia, hypercholesterolemia, and gastrointestinal disease, and it has few negative effects.^[20]^ It is a natural therapy consistent with the principles of obesity therapy of the World Health Organization (WHO). Therefore, catgut embedding at acupoints for simple obesity might attain wider clinical applications globally.

An increasing number of reports on catgut embedding at acupoints for simple obesity have appeared in the clinical research literature in recent years. However, the clinical efficacy of this treatment has been unevenly reported, and large-sample, multicenter, randomized controlled clinical trials (RCTs) are lacking. Previous systematic reviews found limited results supporting catgut embedding at acupoints as an effective treatment method for simple obesity.^[2,17,21,22]^ Most of the systematic reviews have ignored the course of treatment of acupoint catgut embedding for the simple obesity, and the confounding factors resulted in bias. Moreover, most of the systematic reviews failed to search the clinical trials registry platform for ongoing trials. According to *Evidence-based Guidelines of Clinical Practice with Acupuncture and Moxibustion for Simple Obesity*, acupuncture treatment for simple obesity has a longer course of treatment and generally must be performed for at least 3 months to achieve stable efficacy.^[23]^ To reduce the heterogeneity of the included literature, we will assess only those trials in which the course of treatment was more than 3 months. This review and meta-analysis will be conducted to objectively evaluate the clinical efficacy of catgut embedding at acupoints for simple obesity and to provide evidence for a better weight loss method. This is the first meta-analysis of acupoint catgut embedding for simple obesity to examine the effects and adverse reactions based on the analysis of RCTs of suitable treatment paradigms.

## 2 Methods

### 2.1 Study registration

Our systematic review protocol is reported according to the Preferred Reporting Items for Systematic Reviews and Meta-Analyses Protocols (PRISMA-P) statement guidelines.^[24]^ The systematic review protocol is registered in INPLASY (2020110045; DOI: 10.37766/inplasy2020.11.0045).

### 2.2 Inclusion criteria for study selection

#### 2.2.1 Types of studies

This review will be limited to RCTs involving a treatment course longer than 3 months and comparing acupoint catgut embedding with other interventions; language and blinding will not be restricted.

#### 2.2.2 Types of participants

The participants in the RCTs must meet the acknowledged diagnostic and inclusion criteria and have a clear diagnosis of simple obesity. Patient characteristics such as gender and race will not be restricted.

#### 2.2.3 Types of interventions

2.2.3.1 Clinical trials in which the treatment group used acupoint catgut embedding as the sole treatment will be included (studies that used diet or exercise therapy in the control group will be allowed).

2.2.3.2. Clinical trials that used other types of acupuncture, drugs, lifestyle modifications (diet or exercise therapy), placebo ,or waitlists as control interventions will be included.

2.2.3.3 Trials involving combinations of acupoint catgut embedding and other acupuncture therapies will be excluded, as will studies comparing different forms of acupoint catgut embedding.

2.2.3.4 The materials used for acupoint catgut embedding will not be considered.

#### 2.2.4 Types of outcomes

##### 2.2.4.1 Primary outcomes

The primary outcomes will consist of the improvement rate and reduction in body weight (BW).

##### 2.2.4.2 Secondary outcomes

The secondary outcomes will be BMI, waist circumference (WC), fat percentage (F %) and adverse effects.

### 2.3 Search methods for identification of studies

A search strategy will be used and conducted according to the Cochrane Handbook guideline.^[25]^ Electronic searches of the Cochrane Library, PubMed, Springer Medline, EMBASE, Web of Science, CNKI, WANFANG, CBM, and VIP databases will be performed. The Chinese Clinical Trial Registry Centre (http://www.chictr.org.cn/) and the WHO International Clinical Trials Registry Platform (ICTRP) (https://clinicaltrials.gov/) will also be searched for ongoing trials. All of the databases will be searched from the available date of inception to August 2020. The key words used will be catgut embedding, catgut implantation, thread implantation, thread embedding, simple obesity, obesity, weight loss, weight control, weight reduction, adiposity, adiposis, and over-weight.

We will use combinations of free words and subject terms to search, which will not be limited by language, and cross-searching of all of the databases will be performed to ensure that all relevant articles are identified. An example search strategy for the PubMed database is shown in Table 1. Similar search strategies will be adopted for the Cochrane Library, Springer Medline, EMBASE, Web of Science databases, WANFANG, CBM, and VIP databases.

**Table 1:** Search strategy in PubMed database.

Number	Search terms
#1	“Obesity”[Mesh]
#2	“Obesity, Abdominal”[Mesh]
#3	body mass index[Title/Abstract] OR visceral adipose tissue[Title/Abstract] OR fat mass[Title/Abstract] OR weight loss[Title/Abstract] OR weight reduction [Title/Abstract] OR weight control[Title/Abstract] OR weight decrease [Title/Abstract] OR overweight[Title/Abstract] OR body weight[Title/Abstract] OR abdominal fat[Title/Abstract]
#4	#1 OR #2 OR #3
#5	thread implantation[Title/Abstract] OR thread embedding[Title/Abstract] OR acupoints catgut embedding[Title/Abstract] OR catgut implantation[Title/Abstract]
#6	“Randomized Controlled Trials as Topic”[Mesh]
#7	“Randomized Controlled Trial” [Publication Type]
#8	“Pragmatic Clinical Trial” [Publication Type]
#9	“Pragmatic Clinical Trials as Topic”[Mesh]
#10	“Intention to Treat Analysis”[Mesh]
#11	“random allocation”[Mesh Terms]
#12	random∗[Title/Abstract]
#13	#6 OR #7 OR #8 OR #9 OR #10 OR #11 OR #12
#14	#4 AND #5 AND #13

### 2.4 Date collection and analysis

#### 2.4.1 Selection of studies

**Figure 1:**
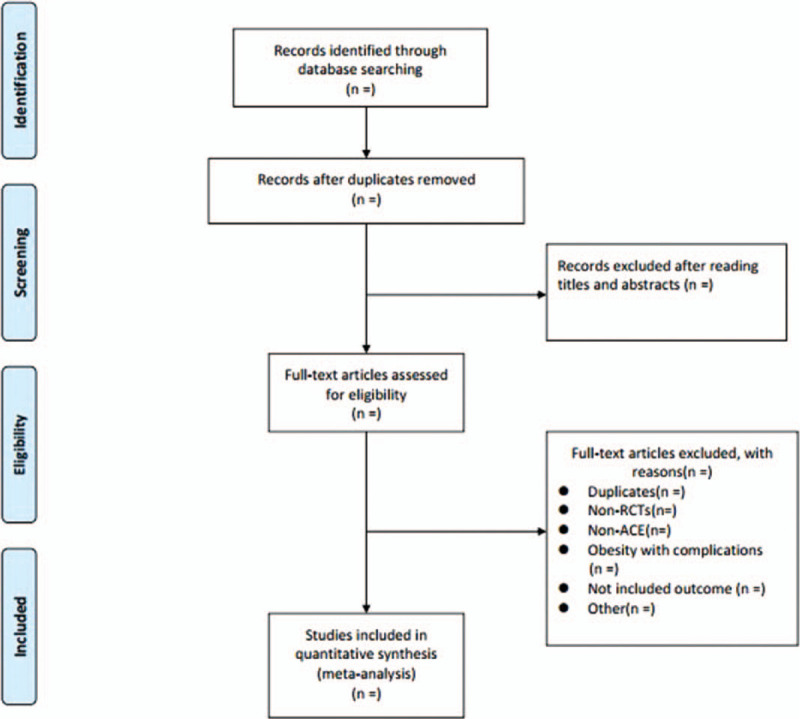
Prisma fow chart.

Literature screening, data extraction and risk of bias evaluation will be performed by 2 reviewers (XC and ZZ) and will be cross-checked. The titles of the studies will be read as the first step in selecting the literature. After excluding non-relevant literature, the abstracts and full texts of the remaining studies will be read to determine inclusion. In cases of duplicate publications, we will select the original publication. A third researcher (WH) will assist in the evaluation in cases of disagreement. Details of the selection process are shown in the PRISMA flow chart (Fig. 1).

#### 2.4.2 Data extraction and management

Two reviewers (LW and SC) will independently complete this step. A standard data extraction form will be used to record the following information:

1. general information on the study (author name(s) and contact information, research location and year of publication);
2. patient characteristics (age, gender, and race) and diagnostic and improvement criteria for obesity;
3. risk of bias assessment;
4. experimental intervention details and control interventions; the revised Standard for Reporting Interventions in Clinical Trials of Acupuncture (STRICTA)^[26]^ will be used in conjunction with CONSORT^[27]^ to extract the details;
5. outcome data, adverse effects and follow-up. NoteExpress software will be used to help the reviewers manage data and identify duplicate publications. When 2 or more reports describe a single trial, only 1 publication will be included.

#### 2.4.3 Assessment of risk of bias in included studies

The risk of bias in the included studies will be assessed according to the Cochrane Collaboration Risk of Bias Tool.^[28]^ The methodology will be evaluated according to the following domains: random sequence generation, allocation concealment, blinding of participants and personnel, blinding of outcome assessment, incomplete outcome data, selective reporting, and other bias. Two reviewers (LW and HW) will categorize each trial as low risk, high risk or unclear risk. A third reviewer (XC) will assist in resolving the disagreements.

#### 2.4.4 Measures of treatment effect

RevMan software (version 5.3 for Windows; the Nordic Cochrane Centre, Copenhagen, Denmark) will be implemented for statistical analysis. Forest plots will be used to illustrate the relative strength of curative effects. The relative risk (RR) will be used as the index for dichotomous data, and the mean difference (MD) will be used as the effect index for continuous variables. The estimated value and 95% CI of each effect will be calculated.

#### 2.4.5 Missing data

The corresponding author will be contacted to obtain missing data. If missing data cannot be obtained, the study will be excluded.

#### 2.4.6 Assessment of heterogeneity

The heterogeneity among included studies will be determined using the χ^2^ test (test level α = 0.1) and the *I*^2^ statistic.^[29]^ The fixed effect model or random effects model will be chosen depending on the *I*^2^ statistic. Obvious clinical heterogeneity will be addressed by conducting subgroup analysis or sensitivity analysis or by conducting descriptive analysis alone after assessing the reasons.^[30]^

#### 2.4.7 Assessment of reporting biases

When 10 or more trials are included in a meta-analysis, Funnel plots will be used to illustrate publication bias.^[31]^

#### 2.4.8 Data synthesis

RevMan (V.5.3) will be used to calculate the RR for dichotomous data and the MD for continuous variables. The estimated value and 95% CI of each effect will be calculated. If the research results are not significantly different (*I*^2^ ≤ 50%), a fixed effect model will be used for the meta-analysis. If the research results are significantly different (*I*^2^ > 50%), a meta-analysis will be performed using a random effects model after further analysis of the heterogeneity of the sources. If the data are not suitable to quantitatively combine, text will be provided to summarize the findings of the included publications. For trials reporting only pre- and post-intervention values, the mean changes will be obtained by subtracting the pre-measurements from the post-measurements. Accordingly, the standard deviation (s.d.) for changes will be estimated.^[32]^

#### 2.4.9 Subgroup analysis

The basis of the subgroup will be the type of control group and the frequency of treatment.

#### 2.4.10 Sensitivity analysis

If the test for heterogeneity remains at a value of *P* < .1 after subgroup analysis, a sensitivity analysis will be performed. The low quality studies will be excluded, and the meta-analysis will be performed again.

#### 2.4.11 Quality of evidence

The researchers will evaluate the quality of evidence with the Grading of Recommendations, Assessment, Development, and Evaluation (GRADE). The quality of evidence will be rated as very low, low, moderate, or high.

## 3 Discussion

A network meta-analysis of acupuncture and related therapies for obesity^[33]^ in 2018 suggested that acupoint catgut embedding is more effective than manual acupuncture. For people with simple obesity in China, acupuncture treatment should be performed every day or every other day. Due to personal and work demands, patient compliance with physician instructions can be difficult. Catgut embedding at acupoints involves a different pattern of treatment from other acupuncture methods; some embedded materials can stimulate acupoints for 2 to 4 weeks, which greatly increases patient compliance.^[2]^ As clinical studies have shown,^[34]^ the treatment costs of acupoint catgut embedding are lower than those of electroacupuncture. In selecting weight loss treatment options for patients, in addition to considering efficacy, it is important to consider the patient's economic situation. To optimize the use of medical resources, low-cost treatments of strong therapeutic effect are desirable.

This is a protocol for a systematic review that aims to evaluate the treatment effects and the adverse reactions associated with acupoint catgut embedding therapy to treat simple obesity. The limitation of this study is that the differences in selecting embedding acupoints in each trial and the variable methodology in included studies may result in heterogeneity. Despite this limitation, this review will be the first quantitative analysis of acupoint catgut embedding therapy involving a treatment course of more than 3 months, and treatment advice will be provided. We hope that this systematic review will help with decision making in the clinical treatment of simple obesity.

However, this study has several limitations. One limitation is that the embedding acupoints may be not unified and the poor quality of methodologies in the included studies may introduced confounding factors. Another is that positive results are more likely to be published in the literature, which may have introduced publication bias in the present study.

## Author contributions

Wei Huang, Xia Chen and Yanji Zhang have contributed equally to this work. All authors have read and approved the publication of the protocol.

**Conceptualization:** Wei Huang, Xia Chen, Zhongyu Zhou.

**Data curation:** Dan Wei, Yanji Zhang, Xia Chen, Lihua Wang, Yingrong Zhang.

**Formal analysis:** Zhongyu Zhou, Wei Huang, Dan Wei.

**Investigation:** Yanji Zhang, Zhongyu Zhou, Dan Wei.

**Methodology:** Xia Chen, Wei Huang, Jiajie Wang.

**Software:** Yanji Zhang, Lihua Wang, Jiajie Wang, Yingrong Zhang.

**Supervision:** Zhongyu Zhou, Wei Huang.

**Writing – original draft:** Wei Huang, Xia Chen, Yanji Zhang.

**Writing – review & editing:** Zhongyu Zhou, Wei Huang.
